# Surgical Removal of an Impacted Palatal Mesiodens Prior to Orthodontic Treatment: A Case Report

**DOI:** 10.7759/cureus.112617

**Published:** 2026-07-13

**Authors:** Allan E Sanabria Marcia, Flavio Sanabria, Francy E Rojas Figueroa, Carla D Ceballos Vivas, Maria C Sayago Montesdeoca

**Affiliations:** 1 Dentistry, Excel Dental, Lynn, USA; 2 Dentistry, General Practice, Choluteca, HND; 3 Dentistry, Clinica Dentisa SAS, Bogota, COL; 4 Dentistry, Conway Dental Care, Orlando, USA; 5 Dentistry, General Practice, Tallahassee, USA

**Keywords:** adolescent, cbct, cone-beam computed tomography, impacted tooth, mesiodens, oral surgery, orthodontic treatment, palatal impaction, supernumerary tooth, surgical extraction

## Abstract

An impacted mesiodens is the most common type of supernumerary tooth and may interfere with the eruption, alignment, and orthodontic movement of permanent maxillary incisors. Accurate localization is essential for safe surgical planning and minimizing damage to adjacent anatomical structures. This case report describes the diagnosis and surgical management of a palatally impacted mesiodens in a 14-year-old male patient referred for orthodontic treatment because of dental crowding and malalignment. Routine panoramic radiography revealed an impacted supernumerary tooth in the anterior maxilla, and cone-beam computed tomography (CBCT) was subsequently performed to determine its precise three-dimensional position. CBCT demonstrated the mesiodens located palatal to the root of the maxillary right central incisor. Following discussion with the patient's parent and informed consent, surgical extraction was performed under local anesthesia using a palatal envelope flap. The impacted tooth was successfully exposed, carefully removed, and the surgical site was irrigated and closed with 3-0 chromic gut sutures. The procedure was completed without complications, and postoperative healing was uneventful, allowing the patient to proceed with orthodontic treatment. This case highlights the importance of CBCT-guided surgical planning and timely removal of an impacted mesiodens before orthodontic therapy to facilitate predictable treatment outcomes while preserving adjacent structures.

## Introduction

Supernumerary teeth are developmental anomalies characterized by the presence of teeth in excess of the normal dentition. Mesiodens, located in the maxillary midline between the central incisors, is the most common type of supernumerary tooth and is frequently identified during routine radiographic examinations in children and adolescents [1-4].

Accurate localization of impacted mesiodens is essential for appropriate treatment planning. Although panoramic radiography is commonly used for initial detection, cone-beam computed tomography (CBCT) provides three-dimensional (3D) visualization of the impacted tooth and its relationship to adjacent anatomical structures, allowing precise surgical planning while minimizing the risk of injury to neighboring teeth and supporting tissues [5-8].

Early surgical removal is generally recommended when an impacted mesiodens is expected to interfere with normal eruption, orthodontic tooth movement, or occlusal development. Timely intervention before orthodontic treatment eliminates mechanical obstacles, facilitates alignment of the permanent dentition, and improves the predictability of orthodontic treatment outcomes [9-12].

This case report describes the diagnosis, CBCT-guided localization, and successful surgical removal of a palatally impacted mesiodens in a healthy adolescent male patient before orthodontic treatment. The case highlights the importance of advanced 3D imaging, meticulous surgical technique, and interdisciplinary collaboration in achieving predictable surgical and orthodontic outcomes [13,14].

## Case presentation

A healthy 14-year-old male patient presented for orthodontic evaluation because of dental crowding and malalignment of the maxillary anterior teeth. His medical history was noncontributory, with no history of systemic disease, facial trauma, medication use, or known allergies. Clinical examination revealed anterior maxillary crowding without swelling, pain, or signs of infection.

As part of the routine orthodontic assessment, a panoramic radiograph was obtained and revealed an impacted supernumerary tooth in the anterior maxilla (Figure 1). To accurately determine the 3D position of the impacted tooth and its relationship to the adjacent permanent incisors, CBCT was performed (Figure 2). The CBCT examination demonstrated a palatally impacted mesiodens located immediately posterior to the root of the maxillary right central incisor (tooth #8). The mesiodens exhibited complete root formation, with no evidence of root resorption involving the adjacent teeth or of associated cystic pathology.

**Figure 1:**
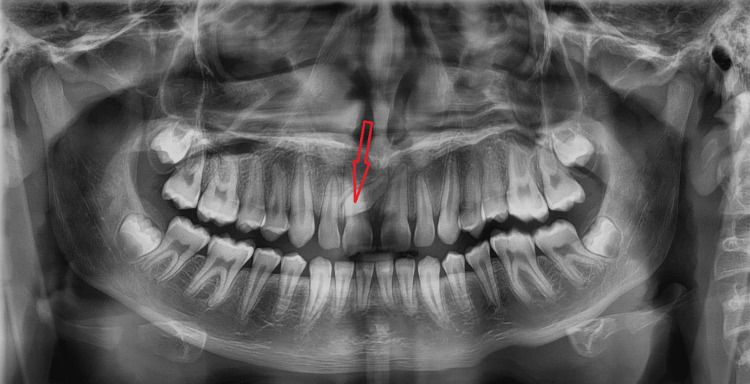
Panoramic radiograph demonstrating the impacted mesiodens in the anterior maxilla during the initial orthodontic evaluation The red arrow indicates the impacted supernumerary tooth located between the maxillary central incisors.

**Figure 2:**
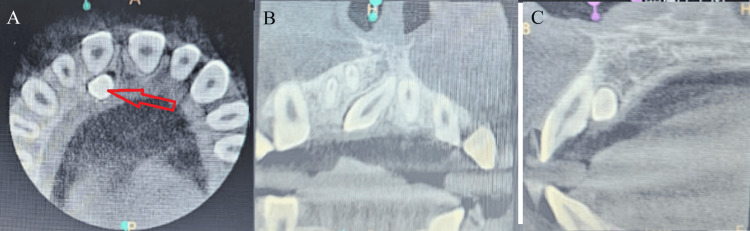
Cone-beam computed tomography demonstrating the three-dimensional position of the impacted mesiodens. (A) Axial view, (B) Sagittal view, and (C) Coronal view. The red arrow identifies the palatally impacted supernumerary tooth located immediately posterior to the maxillary right central incisor (tooth #8), providing detailed visualization of its relationship to the adjacent permanent incisors for surgical planning.

Because the impacted mesiodens represented a potential obstacle to orthodontic tooth movement, surgical removal before initiation of orthodontic treatment was recommended. The diagnosis, treatment options, potential risks, benefits, and possible complications were thoroughly discussed with the patient's mother, who provided written informed consent for the procedure.

The patient was referred to an oral surgeon for surgical extraction of the impacted mesiodens. Local anesthesia was achieved using 2% lidocaine with 1:100,000 epinephrine administered by buccal infiltration, incisive nerve block, and greater palatine nerve block. A palatal crevicular (envelope) flap was designed using an intrasulcular incision without vertical releasing incisions. A full-thickness mucoperiosteal flap was carefully elevated to expose the underlying palatal cortical bone (Figure 3).

**Figure 3:**
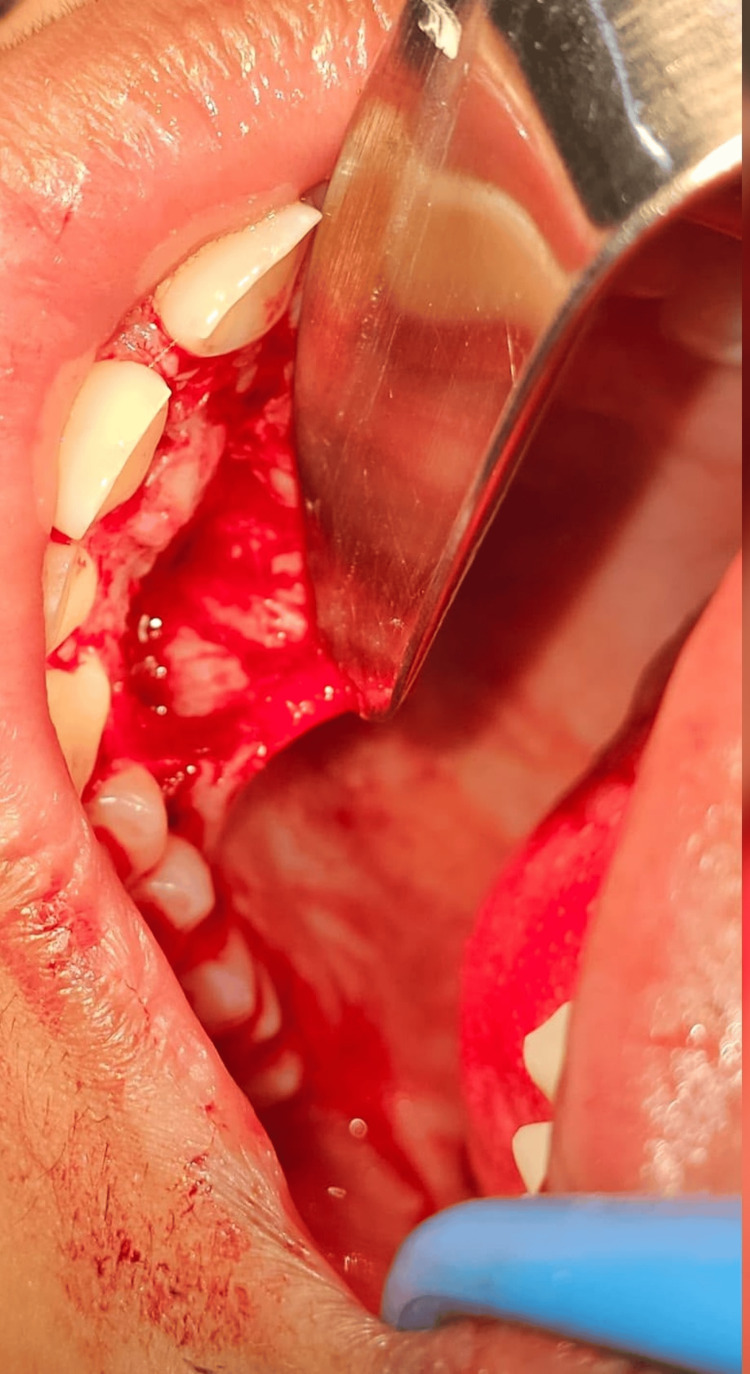
Elevation of the palatal envelope flap. Intraoperative photograph demonstrating elevation of a full thickness palatal mucoperiosteal (envelope) flap, providing surgical access to the underlying palatal cortical bone for exposure of the impacted mesiodens.

Following conservative removal of the overlying palatal cortical bone under copious sterile saline irrigation, the impacted mesiodens was identified (Figure 4). The tooth was carefully luxated and extracted while preserving the surrounding alveolar bone and adjacent permanent teeth (Figure 5). The extracted mesiodens is shown in Figure 6. The surgical site was then inspected to confirm complete removal of the supernumerary tooth without residual fragments, thoroughly irrigated with sterile physiologic saline, and prepared for flap repositioning. 

**Figure 4:**
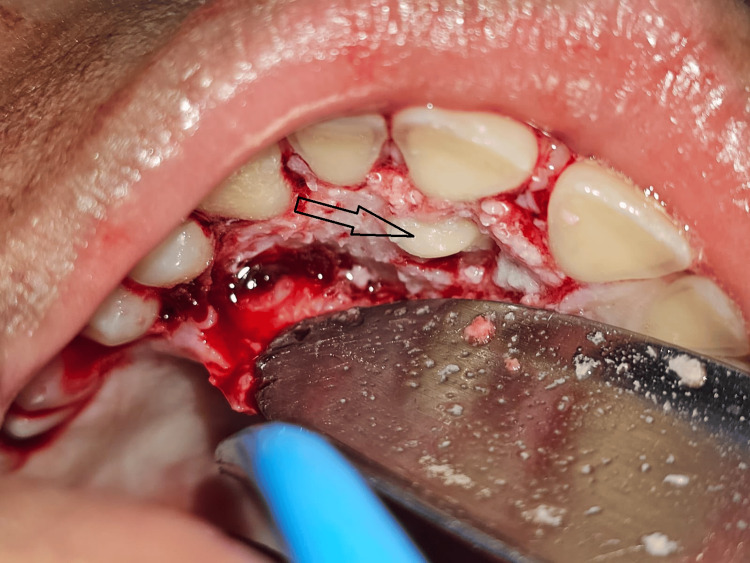
Surgical exposure of the impacted mesiodens. Intraoperative clinical photograph demonstrating surgical exposure of the impacted mesiodens following conservative removal of the overlying palatal cortical bone. The black arrow indicates the crown of the impacted mesiodens, which is clearly visible after flap reflection and osteotomy, allowing controlled luxation and subsequent extraction while preserving the adjacent permanent teeth.

**Figure 5:**
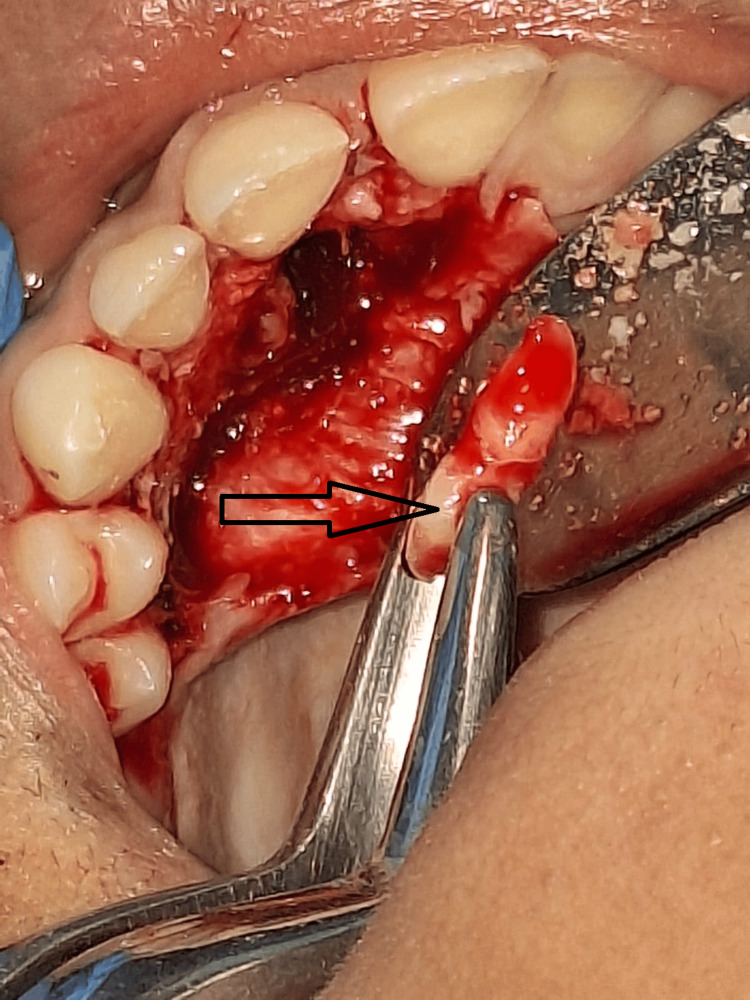
Extraction of the impacted mesiodens. Intraoperative clinical photograph demonstrating removal of the impacted mesiodens following complete surgical exposure. The black arrow indicates the impacted mesiodens as it is being carefully extracted with forceps while preserving the surrounding alveolar bone and adjacent permanent teeth.

**Figure 6:**
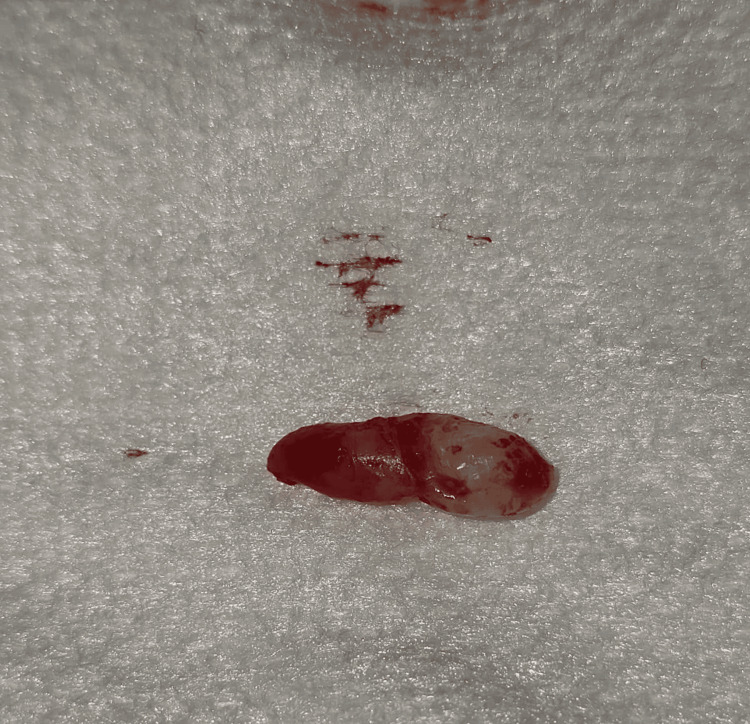
Extracted mesiodens following surgical removal. The supernumerary tooth was removed intact without damage to the adjacent permanent dentition or surrounding alveolar bone.

Primary closure was achieved using interrupted 3-0 chromic gut sutures, providing tension-free adaptation of the palatal flap to promote favorable soft tissue healing (Figure 7). The patient tolerated the procedure well without intraoperative or immediate postoperative complications. Clinical evaluation demonstrated satisfactory soft tissue closure and preservation of the adjacent dentition, allowing the patient to proceed with the planned orthodontic treatment following an appropriate healing period. 

**Figure 7:**
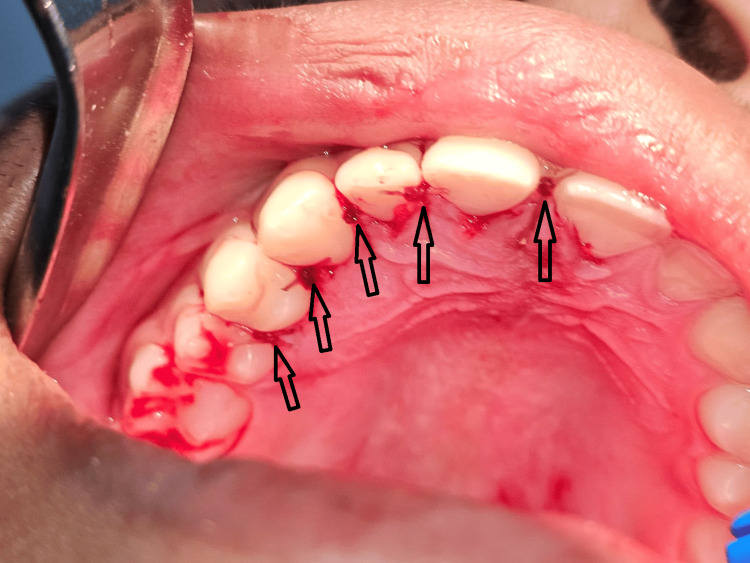
Final tension-free primary closure of the palatal surgical site following mesiodens extraction. Clinical intraoral photograph showing the repositioned palatal flap after extraction of the impacted mesiodens. The arrows indicate interrupted 3-0 chromic gut sutures providing tension free adaptation of the surgical wound to promote favorable postoperative healing.

## Discussion

Mesiodens is the most common type of supernumerary tooth and is typically located in the maxillary midline. Although many cases are discovered incidentally during routine dental examinations, impacted mesiodens may interfere with the eruption and alignment of the permanent maxillary incisors, contribute to dental crowding, delay orthodontic treatment, and occasionally result in complications such as root resorption, midline diastema, or dentigerous cyst formation. Early diagnosis is therefore essential to prevent long-term functional and esthetic complications while facilitating timely interdisciplinary treatment [1-4].

Conventional panoramic radiography is commonly used as the initial imaging modality for detecting impacted supernumerary teeth. However, panoramic images provide only two-dimensional information and may not accurately demonstrate the spatial relationship between the impacted tooth and adjacent anatomical structures. In the present case, CBCT was essential for accurately determining the 3D position of the mesiodens relative to the maxillary right central incisor (tooth #8). This additional diagnostic information facilitated precise surgical planning, minimized unnecessary bone removal, and reduced the risk of injury to the adjacent permanent teeth [5-9].

The timing of surgical removal of an impacted mesiodens remains an important clinical consideration. Most authors recommend extraction when the supernumerary tooth interferes with eruption, orthodontic treatment, or normal occlusal development [10-12]. In the present patient, removal before initiation of orthodontic therapy eliminated a mechanical obstacle that could have compromised tooth movement and treatment efficiency. Early intervention also simplified surgical access while preserving the surrounding alveolar bone and adjacent dental structures.

A palatal envelope flap was selected because it provided excellent surgical access while preserving the vascular supply and allowing tension-free primary closure. Conservative osteotomy performed under copious sterile saline irrigation enabled safe visualization and removal of the impacted tooth while minimizing trauma to the surrounding tissues. Careful inspection of the surgical site confirmed complete removal of the mesiodens without residual fragments, and primary closure with interrupted 3-0 chromic gut sutures promoted favorable soft tissue healing. No intraoperative or immediate postoperative complications were observed [13].

This case also highlights the importance of interdisciplinary collaboration between the orthodontist and oral surgeon. Routine orthodontic imaging enabled early detection of the impacted mesiodens, while CBCT-guided surgical planning facilitated safe removal before orthodontic treatment. Such coordinated management minimizes surgical risk, prevents delays in orthodontic therapy, and improves the predictability of overall treatment outcomes [14].

Although this report describes the successful management of a single patient, it demonstrates the clinical value of combining advanced 3D imaging with meticulous surgical technique for the management of impacted mesiodens. Early CBCT-guided diagnosis and timely surgical intervention can facilitate safe removal, preserve adjacent anatomical structures, and optimize subsequent orthodontic treatment. Additional prospective studies with larger patient populations are warranted to further evaluate long-term surgical and orthodontic outcomes following early intervention.

## Conclusions

Early identification of impacted mesiodens is essential for preventing complications that may interfere with normal dental development and orthodontic treatment. In this case, CBCT provided precise 3D localization of the impacted tooth, enabling accurate surgical planning and safe removal while preserving the adjacent teeth and surrounding anatomical structures. Surgical extraction before orthodontic therapy eliminated a potential obstacle to tooth movement and resulted in an uneventful postoperative recovery. This case highlights the importance of CBCT-guided diagnosis, meticulous surgical technique, and interdisciplinary collaboration between oral surgery and orthodontics in achieving predictable surgical and orthodontic outcomes.

## References

[1] Van Buggenhout G, Bailleul-Forestier I (2008). Mesiodens. Eur J Med Genet.

[2] Kim H, Song JS, Shin TJ, Kim YJ, Kim JW, Jang KT, Hyun HK (2024). Image segmentation of impacted mesiodens using deep learning. J Clin Pediatr Dent.

[3] Davidson CL, Smit C, Nel S (2025). Supernumerary teeth: a pictorial review and revised classification. J Oral Biol Craniofac Res.

[4] Nanu DP, Favre NM, Walsh J, Farsar C, Le TA, Carr MM (2023). Palatal supernumerary tooth in a pediatric patient. Cureus.

[5] Jang Y, Lee J, Kim G (2025). Application of a surgical guide for the extraction of impacted mesiodens: a case series. J Clin Pediatr Dent.

[6] Pasaco González JA, Luzuriaga Torres YD, Calderón Calle ME (2023). Surgical approach techniques for extraction of impacted or retained mesiodens: literature review. World J Adv Res Rev.

[7] Goksel S, Agirgol E, Karabas HC, Ozcan I (2018). Evaluation of prevalence and positions of mesiodens using cone-beam computed tomography. J Oral Maxillofac Res.

[8] Kumar KR, Varsha S, Kumar RM, Kumar BJ, Pal S, Handique S (2024). Orthodontic-surgical management of triple impacted mesiodens and maxillary incisors: a case report. Ann Maxillofac Surg.

[9] Avci B, Kaya S (2025). Impact of mesiodens on transverse jaw dimension and nasal septum deviation in pediatric patients: a retrospective CBCT study. BMC Oral Health.

[10] Adachi M (2026). Mesiodens removal: resolving the early versus late intervention dilemma. J Oral Maxillofac Surg.

[11] Boukhris H, Zidani H, Ben Haj Salah K, Bouslama G, Ben Youssef S (2025). Mesiodens: a multidisciplinary approach to diagnosis and management. J Dent Health Oral Disord Ther.

[12] Mazzocchi A, Mazzocchi S, Floria G (2026). Clinical and timing considerations for surgical removal of mesiodens: early vs late treatment. SVOA Dent.

[13] Sharifi R, Shafiei S, Moslemi H, Mohammadi Khah M (2021). Intraoral transnasal approach for surgical extraction of bilateral deeply impacted mesiodens: a case report. Clin Case Rep.

[14] Ata-Ali F, Ata-Ali J, Peñarrocha-Oltra D, Peñarrocha-Diago M (2014). Prevalence, etiology, diagnosis, treatment and complications of supernumerary teeth. J Clin Exp Dent.

